# The tubulin database: Linking mutations, modifications, ligands and local interactions

**DOI:** 10.1371/journal.pone.0295279

**Published:** 2023-12-08

**Authors:** Izra Abbaali, Danny Truong, Shania Deon Day, Faliha Mushayeed, Bhargavi Ganesh, Nancy Haro-Ramirez, Juliet Isles, Hindol Nag, Catherine Pham, Priya Shah, Ishaan Tomar, Carolina Manel-Romero, Naomi S. Morrissette

**Affiliations:** Department of Molecular Biology and Biochemistry, University of California, Irvine, CA, United States of America; Centre de Recherche en Biologie cellulaire de Montpellier, FRANCE

## Abstract

Microtubules are polymeric filaments, constructed of α-β tubulin heterodimers that underlie critical subcellular structures in eukaryotic organisms. Four homologous proteins (γ-, δ-, ε- and ζ-tubulin) additionally contribute to specialized microtubule functions. Although there is an immense volume of publicly available data pertaining to tubulins, it is difficult to assimilate all potentially relevant information across diverse organisms, isotypes, and categories of data. We previously assembled an extensive web-based catalogue of published missense mutations to tubulins with >1,500 entries that each document a specific substitution to a discrete tubulin, the species where the mutation was described and the associated phenotype with hyperlinks to the amino acid sequence and citation(s) for research. This report describes a significant update and expansion of our online resource (TubulinDB.bio.uci.edu) to nearly 18,000 entries. It now encompasses a cross-referenced catalog of post-translational modifications (PTMs) to tubulin drawn from public datasets, primary literature, and predictive algorithms. In addition, tubulin protein structures were used to define local interactions with bound ligands (GTP, GDP and diverse microtubule-targeting agents) and amino acids at the intradimer interface, within the microtubule lattice and with associated proteins. To effectively cross-reference these datasets, we established a universal tubulin numbering system to map entries into a common framework that accommodates specific insertions and deletions to tubulins. Indexing and cross-referencing permitted us to discern previously unappreciated patterns. We describe previously unlinked observations of loss of PTM sites in the context of cancer cells and tubulinopathies. Similarly, we expanded the set of clinical substitutions that may compromise MAP or microtubule-motor interactions by collecting tubulin missense mutations that alter amino acids at the interface with dynein and doublecortin. By expanding the database as a curated resource, we hope to relate model organism data to clinical findings of pathogenic tubulin variants. Ultimately, we aim to aid researchers in hypothesis generation and design of studies to dissect tubulin function.

## Introduction

Microtubules are essential to all eukaryotes, where they underpin critical structures such as spindles, centrosomes, axonemes, and cytoplasmic arrays. Microtubules and microtubule-associated structures are assembled from a superfamily of related proteins known as tubulins [1–3]. The tubulin superfamily consists of six families each comprised of tubulins with greater similarity and comparable functions [4]. While α- and β-tubulins form heterodimers that assemble into microtubules, γ-, δ-, ε- and ζ-tubulins contribute to specialized microtubule structures. All eukaryotes have genes for α-, β- or γ-tubulins, and may have more than one gene for these tubulins, known as isoforms or isotypes. Differences in amino acids between isotypes modify biochemical properties to fine-tune cell-specific requirements [5]. δ-, ε- and/or ζ-tubulins are specified by single genes that are found in organisms that construct appendage-containing centrioles [4,6]. δ-, ε- and ζ-tubulin genes are missing (lost) from organisms that lack centrioles (higher land plants and most fungi) or organisms with atypical centrioles such as *Caenorhabditis elegans* and *Drosophila melanogaster*. δ- and ζ-tubulins are thought to be evolutionarily interchangeable, with ζ-tubulin genes found in various marine invertebrates, diverse protozoa, marsupials, reptiles, amphibians, and fish, but absent from placental mammals, including humans [4,6].

Tubulins are extraordinarily conserved across diverse eukaryotic lineages because functional constraints restrict sequence diversity. They must maintain essential interactions with GTP, as well as a multitude of proteins, including other tubulins, motors, and microtubule-associated proteins for proper function. Consequently, sequence identity and overall structural features are conserved within and across tubulin families. For example, α- and β-tubulins share ~40% identity and 60% similarity. This translates into conserved topology: α- and β-tubulin monomers have highly similar structures that consist of three subdomains: a GTP binding N-terminal region, a central region and a C-terminal region that contributes to the microtubule surface bound by microtubule-associated proteins (MAPs) and motor proteins [7,8]. Each globular monomer is comprised of a core formed from two beta sheets enveloped by external alpha-helices. Although the structure of γ-tubulin is similar, γ-tubulins have conserved insertions and deletions (indels) relative to α- and β-tubulins that may contribute to specialized interactions [9]. Laterally associated γ-tubulins in the γ-tubulin ring complex organize into a “lock washer-like structure” that provides a template for microtubule nucleation [10]. To date, there are no available experimentally determined structures for δ-, ε- and ζ-tubulins, although alignments and threading have illuminated key conserved regions as well as distinct areas for these tubulin families [6,11]. Most recently, computational methods that predict structures with atomic accuracy were used to model several δ- and ε-tubulins structures which have been deposited in AlphaFold DB [12].

Distinct expression of individual α-, β-, and γ-tubulin isotypes during development or in a cell-specific context contributes to fine-tuning tubulin populations to meet specialized demands for microtubule properties [13]. Vertebrates and many land plants express between six and ten α- and β-tubulin isotypes, each with characteristic expression levels in discrete cell types. Individual isotypes may be critical to cell-specific functions. For example, the unusual α- and β-tubulin isotypes encoded by *mec-12* and *mec-7* are required for touch receptor neuron function in *C*. *elegans* [14,15]. Similarly, a *Drosophila* testes-specific β-tubulin isotype (βTub85D) is specifically required during spermatogenesis to form flagellar axonemes [16]. Small amino acid differences between tubulins confer phylogenetically restricted or isotype-specific sensitivity to microtubule targeting agents (MTAs). Benzimidazoles selectively target fungal and helminth tubulins and dinitroanilines selectively target plant and protozoan tubulins [17,18]. Likewise, in human cancers, upregulated expression of less sensitive tubulin isotypes can confer paclitaxel resistance without tubulin point mutations [19].

Microtubule populations are tuned to best suit developmental or cell-specific demands by adjusting the “tubulin code” which consists of expression of particular α-, β-, and γ-tubulin isotypes accompanied by dynamic PTMs to license microtubule subsets for specific roles [20–23]. The greatest differences between tubulins occur in their carboxy-terminal tails (CTTs) which extend away from these predominantly globular proteins and from the microtubule surface to interact with associated proteins. Both CTTs and other accessible domains can be modified by a variety of PTMs to modulate biochemical properties. Most modifications are dynamic and reversible so can be employed to temporarily mark tubulin subpopulations. Documented PTMs include acetylation, phosphorylation, glutamylation, glycylation, methylation, palmitoylation, succinylation, malonylation, citrullination, MARylation, tyrosination, and detyrosination [22,24–26]. Many PTMs influence the interaction of microtubules with MAPS and motors. In turn, this can influence microtubule stability. For example, detyrosination (removal of the C-terminal tyrosine) does not inherently influence microtubule stability but alters association of depolymerizing kinesin motor proteins with microtubules [27–29]. The α- and β-tubulin CTTs can be modified by the addition of one or more glutamate or glycine residues (polyglutamylation and polyglycylation) to glutamic acid residues [25,30–32]. Microtubules in the mitotic spindle, neuronal projections, axonemes, centrioles and basal bodies are polyglutamylated while axoneme microtubules are the primary site of polyglycylation. Unlike many PTMs, acetylation of α-tubulin K40 is distinct because it localizes to the inside of the microtubule lumen, as does the modifying enzyme αTAT1 [33]. αK40 acetylation is a feature of stable and long-lived microtubules, such as in axonemes, and protects microtubule disassembly induced by depolymerizing MTAs such as colchicine [34,35]. Structural and computational studies indicate that αK40 acetylation alters protofilament interactions to improve microtubule flexibility and resilience to disruption [36]. The role of additional PTMs remains less well-defined, although individual studies hint at critical functions for these alterations.

Tubulin and microtubule structures have been investigated for over twenty years using electron diffraction of zinc-induced tubulin sheets, crystallography of tubulin-stathmin complexes, and helical reconstruction of microtubules imaged in ice [9,37–42]. Recent advances in tubulin expression systems have permitted purification of single isotype dimers and technical and experimental innovations have steadily improved the resolution of tubulin structures [38,42,43]. The ability to co-crystalize MTAs with tubulin has led to key binding pocket information for established and investigational tubulin-targeting drugs [44–47]. Structural information has been incorporated into computational approaches to model conformation changes associated with GTP hydrolysis and drug binding, assembly and catastrophe and the effects of PTMs on the microtubule lattice [36,48–51].

Beginning in the 1970’s, geneticists began to isolate organisms harboring tubulin point mutations that confer temperature sensitive growth, developmental defects, or resistance to MTAs [52–56]. This work was followed by studies that exploited genetically tractable model organisms with directed missense mutations to tubulins [57–62]. As both microtubule stabilizing and destabilizing drugs are used in medicinal contexts, an extensive literature describes tubulin mutations that confer resistance to drugs, including those used to treat cancers or helminth and fungal infections [52,54–56,63–68]. Finally, over the last decade, researchers have identified dominant somatic variants in human tubulin genes that arise during development and underlie neurological defects known as tubulinopathies [69–77]. Collectively, these efforts produce an enormous and ever-increasing body of literature describing the consequences of altered tubulin proteins in a wide variety of organisms. In nearly all cases, missense mutations provide more nuanced information than null or knockout studies of tubulin loci because substitutions modulate phenotypic properties rather than eliminating the protein altogether which may be lethal or without consequence, depending on gene redundancy. For example, the A180T substitution to α-tubulin *tua4* induces aberrant stem and leaf spiraling in *Arabidopsis thaliana*. Genetic suppressors of this dominant missense phenotype include introduction of an early stop mutation that eliminates protein expression [78]. Since several α-tubulin isotypes are co-expressed in *Arabidopsis*, loss of protein specified by the *tua4* locus is without an overt phenotype in marked contrast to expression of the A180T mutant protein.

**Fig 1:**
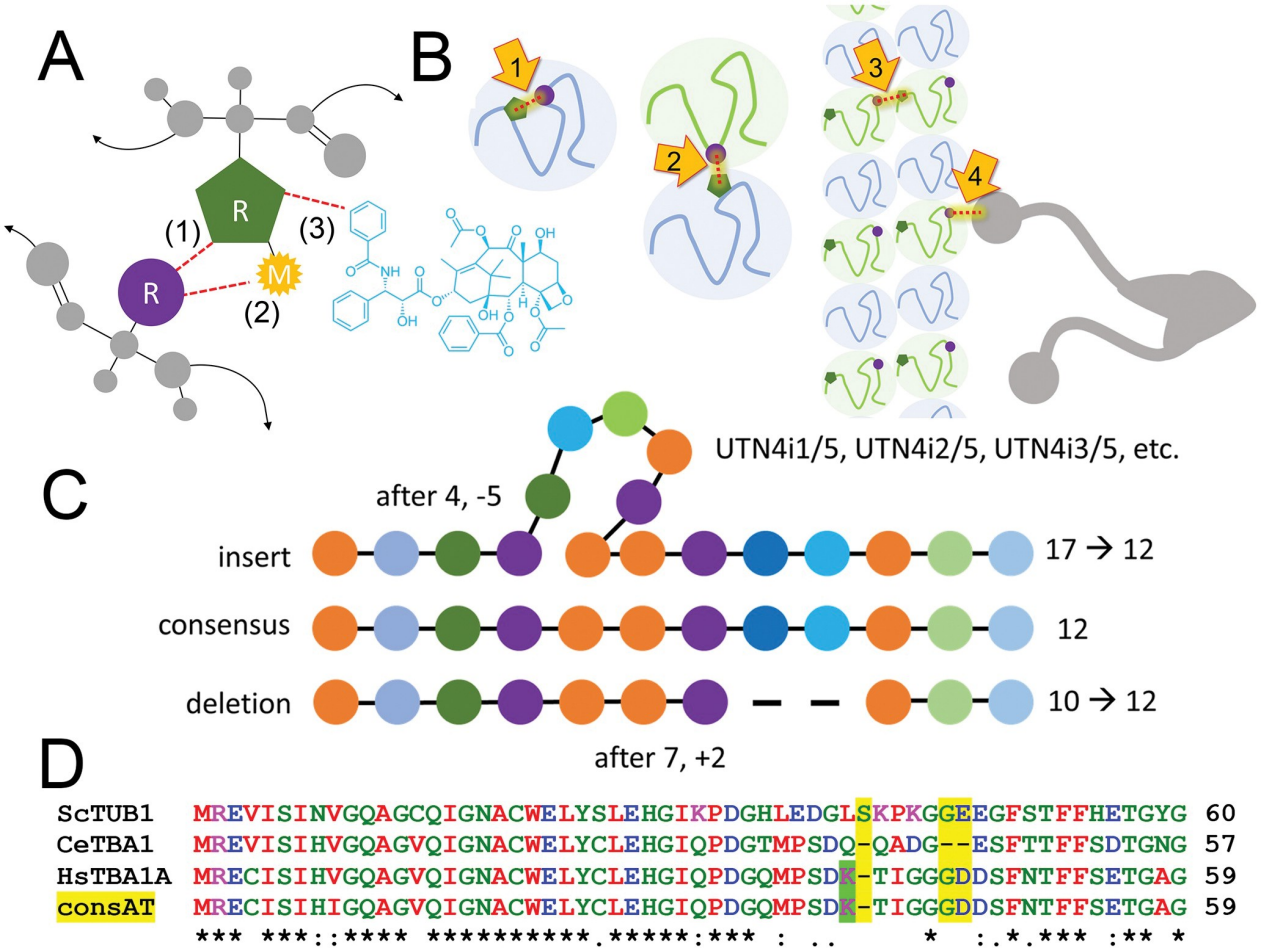
(A) A schematic of an individual sidechain (R) in tubulin (green) illustrates elements that may be altered by a point mutation that changes the amino acid at this position. This may alter: (1) local interactions with other amino acids (purple); (2) the capacity for reversible posttranslational modifications (M) such as phosphorylation or acetylation; and (3) interactions with small molecules including drugs such as paclitaxel (blue). (B) Amino acid interactions encompass: (1) interactions within an individual α- or β- subunit, (2) protein-protein interactions between monomers to form the α-β heterodimer interface; (3) dimer assembly into the microtubule lattice; and (4) interactions with other proteins such as microtubule motors. (C) A schematic illustrates how the universal numbering accommodates insertions or deletions relative to the reference sequence when aligned with homologous sequences harboring insertions or deletions. Amino acid insertions are denoted by “i” following the last conserved residue. This corrects numbering following the insertion to be consistent with the consensus sequence. Similarly, in the case that a tubulin harbors a deletion, the numbering at this site is modified to renumber the following amino acids to be in line with the consensus. (D) The α-tubulin H1’-S2 loop has variation in length as illustrated by an alignment of the consensus sequence with *S*. *cerevisiae* tubulin (+1) and *C*. *elegans* tubulin (-2) (differences highlighted in yellow). Neither *S*. *cerevisiae* (ScTUB1) nor *C*. *elegans* (CeTBA1) tubulins harbor K40, a conserved residue in many α-tubulins, including human α1a-tubulin (TBA1A, green highlight), which can be modified by acetylation to increase microtubule stability.

We recently assembled a database of published tubulin point mutations, with the purpose of evaluating the roles of specific amino acids in microtubule function [79]. This database revealed that identical substitutions to specific amino acids have been identified in a variety of eukaryotes including budding yeast, protozoa, fruit flies, nematodes, unicellular algae, higher land plants, mice, and humans. While some coincident mutations have clearly comparable consequences, others induce apparently dissimilar phenotypes. Nonetheless, we believe that in many cases, the underlying defects remain the result of similar changes to tubulin biochemistry. After developing the tubulin mutation database, we realized that a correspondingly large volume of biochemical, pharmacological, and structural information on tubulins is available to researchers. Again, it is difficult to assimilate all potentially relevant information across diverse organisms, tubulin isotypes, and data types. For example, specific subsets of amino acids in tubulins coordinate interactions with GDP and GTP (physiological ligands) and MTAs such as paclitaxel or colchicine. Similarly, tubulins are dynamically modulated by diverse PTMs to specific residues (**Fig 1A and 1B**). Point mutations to tubulin modify local interactions with ligands and nearby amino acids and may eliminate PTMs. Ultimately, integration of detailed information describing function-altering mutations, structural features, pharmacological properties, and PTM data, is key to dissect how individual amino acids in tubulins function. We now describe expanding the database to encompass information on PTMs and local amino acid interactions. The material indexed in the tubulin database is freely available on our website at TubulinDB.bio.uci.edu. Most entries document data extracted from peer-reviewed papers or are otherwise embedded in protein structure, genetic variant, or mass spectrometry databases. In all cases, entries include hyperlinks to original references, structures, and datasets. With the expansion of this curated resource, we hope to facilitate analysis of clinical variants and design of experiments to understand tubulin function.

## Results

### A universal numbering system for tubulins

Within individual families, alignments establish that some proteins harbor insertions or deletions relative to most other tubulin sequences. For α-, β-, and γ-tubulins, indels are generally rare and typically shift amino acid numbering by a few positions. However, the complexity of the intersecting data we aim to index necessitates creation of a system to map individual tubulins into a common framework for each tubulin family. We used Clustal Omega and EMBOSS alignment tools to define consensus sequences for the α-, β-, and γ- families using 90 α-tubulins, 90 β-tubulins, and 21 γ-tubulins from land plant, nematode, fungus, protozoan, and vertebrate organisms represented in the genetic databases (see methods and accession numbers below). All sequences are full-length, and isotypes are represented. Non-conserved insertions were removed from the alignments to create a generalized consensus. The position of amino acids in the consensus dictates a universal tubulin numbering (UTN) applied to all members of the specific tubulin family. By using Clustal to align the consensus sequence (**S1 Table**) to an individual tubulin, it is possible to determine whether adjustments to the existing numbering are required. In general, the numbering for most α- and β-tubulins is unaltered. Examples of exceptions in key organisms include *Saccharomyces cerevisiae* α1-tubulin (P09733), *D*. *melanogaster* α-tubulin-4 (αTub67C, P06606), *C*. *elegans* α-tubulin-2 (P34690), *Schizosaccharomyces pombe* α-tubulin-2 (nda2, P04688), and human α-tubulin-like 3 (A6NHL2). Inserts in individual sequences are recorded as following a conserved position but do not shift numbering of downstream homologous sequences (**Fig 1C**). This permits equivalent positions to be indexed across isotypes, species, and individual databases. For example, *S*. *cerevisiae* α1-tubulin harbors a single amino acid insertion (S41) that otherwise shifts the numbering of subsequent amino acids +1 relative to many other α-tubulins (yellow highlight, **Fig 1D**). The α-tubulin consensus sequence is used as a guide to re-number the *S*. *cerevisiae* α-tubulin. In this example S41 becomes 40i1/1 and K42 is renumbered as K41. Because inserts may be larger than a single amino acid, this notation defines insert size as well as position of the amino acid in the insert. In the converse case, *C*. *elegans* α1-tubulin (tba1) has two fewer amino acids than the consensus. Therefore, after G44, all residues are renumbered by +2 (yellow highlight). Every relevant dataset entry includes a universal indexing number based on consensus sequences to simplify cross-referencing intersecting positions to comprehensively identify convergences between point mutations, local interactions and PTMs for all members of a tubulin family. This strategy is not novel; some *S*. *cerevisiae* researchers renumber yeast α-tubulin to correspond to vertebrate tubulins. Our aim is to apply this strategy uniformly to all tubulins that harbor unusual indels. Alignments of δ-, ε- or ζ- tubulins revealed that these families have significantly larger insertions (12 to 32 or more residues) that vary in location and sequence. Additionally, the disproportionately high numbers of sequences for some lineages (e.g., protozoa) may bias consensus sequences. Therefore, we have not created a universal numbering system for δ-, ε- and ζ- tubulin families. Researchers may specifically create pairwise amino acid alignments to assess whether positions are equivalent between individual δ-, ε- or ζ-tubulins. The datasets describing δ-, ε- and ζ-tubulins are considerably smaller, so this is not difficult to accomplish.

### A database of tubulin PTMs

**Fig 2:**
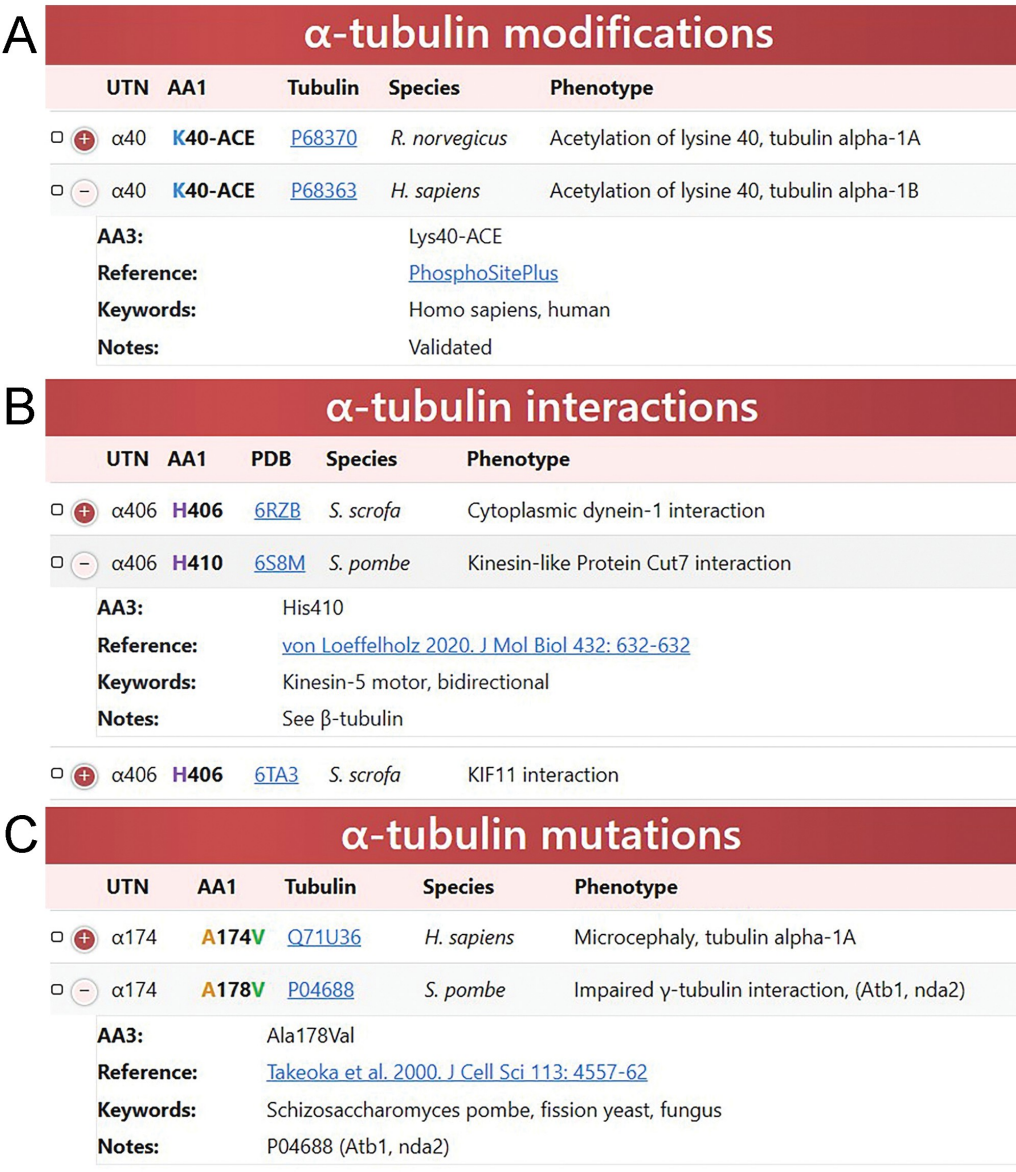
The tubulin database tables. Records can be expanded beyond the single line entry to view additional information and both the tubulin sequence and original reference are hyperlinked (blue). (A) Validated and predicted sites of tubulin PTMs were collected and organized by universal tubulin numbering to document the position and type of the modification; tubulin isotype and species; whether the entry is validated or predicted, with hyperlinks to the tubulin sequence and original data source. (B) Tubulin structures define amino acids that contribute to contours of small molecule binding pockets, interfaces between tubulin subunits in the microtubule lattice and interactions with associated proteins. Individual entries contain hyperlinks to the relevant PDB file and associated publication. This image illustrates the utility of the UTN: amino acid R402 in vertebrates is equivalent to R406 in *S*. *pombe*. This position is located at the interface with microtubule motors. (C) The missense mutations datasets are now organized by universal tubulin numbering. This screenshot shows a previously unappreciated correlation between mutation A174V in human α1-tubulin and A178V in *S*. *pombe*. The fission yeast mutation impairs the interaction of α- and γ-tubulin; the human mutation is associated with a neurodevelopmental disorder.

Tubulin proteins can be covalently modified by enzymes to add small functional groups that modulate microtubule behavior or mark sub-populations for specialized roles [13]. PTMs include acetylation (K), glutamylation (E), glycylation (E), malonylation (K), methylation (H, K, R), nitrosylation (C), palmitoylation (C), phosphorylation (S, T, Y), succinylation (K), SUMOylation (K), ubiquitination (K), O-linked glycosylation (S, T) and CTT de-tyrosination and re-tyrosination. We originally employed PubMed and Google searches to collect information documenting specific PTMs to tubulins in the scientific literature. However, many older studies are insufficiently granular to include the details we require (e.g., amino acid position and specific isotypes modified). Therefore, we moved to collect information using MS and predictive databases. We compiled information to document: (1) the PTM site; (2) the type of modification (phosphorylation, etc.); (3) the modified isotype; (4) species where the modification has been observed; and (5) hyperlinks to the original MS and predictive databases, with the type of data (predicted or validated) noted (**Fig 2A**). Although PTMs are not equally investigated or universally conserved across all isotypes or organisms, intersections of sites of PTMs and mutations that ablate the ability of tubulin to be modified justify investigation of these sites. A key caveat of the PTM entries is that the highly modified CTT tail domains of tubulins are not represented in the MS and predictive datasets used to assemble these entries. While we aim to incorporate entries describing these PTMs into future updates, CTT have much greater heterogeneity and are located beyond the region that is useful for comparative analysis.

### A database of tubulin interactions

The amino acid substitutions described in the tubulin mutation database may modify tubulin function by altering local interactions that influence properties such as contacts with neighboring proteins, MTA binding sites, or regulation by PTMs (**Fig 1**). A 6Å zone was chosen to define the local environment that might be affected by substitutions, PTMs or ligands. We created entries for the 6Å neighbors at the α-β monomer interface and for the dimer interfaces in the microtubule lattice, as well as for microtubule interactions with motors and MAPs (including CAMSAP, doublecortin, EB3, tau, and Stu2). We also defined tubulin residues that are within 6Å of bound ligands such as GTP, GDP and MTAs. This information is organized by individual amino acid in entries that include hyperlinks to the PDB file and original paper (**Fig 2B**).

### Additions to the mutation database

In the interval since we launched the original tubulin mutation database, additional papers have described variants or mutations. The data from these papers provided several hundred new entries. In addition, we collected thousands of entries that describe human tubulin substitutions documented in the ClinVar and BioMuta databases. Although some of these entries coincide with information described in published papers, they only partly overlap, so we chose to maintain all clinical records as stand-alone entries with links to the original sources. We previously noted several intersections of α- and β-tubulin substitutions that are consistent with the conclusion that some tubulinopathy mutations increase or decrease microtubule stability [79]. With expansion of the missense/variant data and UTN indexing, this list is considerably enlarged.

In several model systems, phenotypic or directed screens have identified defects in growth and development that result from altered microtubule stability. When *S*. *cerevisiae* α-tubulin is indexed by the UTN, many entries from a large collection of alanine-scanning mutations [59] clearly overlap with missense mutations from other organisms. Substitutions that increase sensitivity to microtubule-disrupting agents as well as those that induce cold sensitivity are likely to be the consequence of decreased microtubule stability. Alanine substitutions in *S*. *cerevisiae* at α-UTN amino acids R2, E37, D38, E55, D69, H107, D205, R123, D127, T145, D205, R243, D251, R264, E284, R320, R402, R422, or E429 are associated with one or both traits. Loss of the homologous amino acids destabilize microtubules to confer twisting and touch receptor defects in *A*. *thaliana* and *C*. *elegans*, respectively. Moreover, substitutions at many of these conserved positions appear in human tubulinopathies such as lissencephaly [14,80–84]. These phenotypes indicate that these conserved amino acids are critically important for microtubule stability across phyla. UTN indexing allowed us to link the human TUBA1A A174V mutation to the A178V mutation in *S*. *pombe* α1-tubulin (**Fig 2C**). In the context of fission yeast, this substitution blocks the ability of human γ-tubulin to complement deletion of endogenous (*S*. *pombe*) γ-tubulin [85] while A174V in TUBA1A is associated with microcephaly [86]. This suggests that impaired microtubule nucleation may underlie the human phenotype. Consistent with this hypothesis, γ-tubulin substitutions are also associated with malformations of cortical development [87–89].

Convergent loss of conserved residues in β-tubulin also connect basic science observations and clinical findings. Mutations in budding yeast, *C*. *elegans*, and *A*. *thaliana* β-tubulins increase sensitivity to microtubule-disrupting agents, induce touch receptor defects or cause twisting defects, which again suggests that these decrease microtubule stability [15,60,80]. Again, losses of the equivalent amino acids (e.g., R121, E123, R156, T238, P243, G244, Q245, N247, V255, R306, R391, and E401) also appear in clinical conditions. βR318W causes cold-sensitive microtubules in budding yeast [90] and is a *tubb1* variant in cases of human macrothrombocytopenia and congenital hypothyroidism [91–93]. Loss of G98, E205, E410, or D417 in human β-tubulin isotypes is associated with clinical conditions including tubulinopathies (*tubb2b*, *tubb3*, *tubb4a*), oculomotor problems (*tubb3*) and infertility (*tubb8*) [69,75,83, 94–104]. A different set of α- and β-tubulin missense mutations cause benomyl resistance and/or cold stable microtubules in budding yeast [59–61], with parallel losses of conserved amino acids in human clinical conditions. These include α-tubulin positions R214, R215, D218, D306, and R390, as well as β-tubulin positions R46, E108, E123, E194, E198, R241, R282, E288, D295, R318, and C354. While not all equivalent missense mutations yield analogous phenotypes across different organisms or isotypes, identification of key similarities and unanticipated differences in conserved residues should animate additional experiments to define the roles of these amino acids in microtubule function.

The γ-tubulin missense dataset has fewer entries and can be more complex to evaluate than the α- and β-tubulin tables due to the larger size of indels between individual proteins. However, recognition that γ-tubulin mutations lead to malformations of cortical development [87–89] illustrates the importance of further studies in a variety of model organisms. With this update, the UTN indexing revealed several examples of convergent missense mutations. For example, the γ-R72A substitution is lethal in *A*. *nidulans* and *T*. *thermophila* model organisms [58,105] and causes cold sensitivity in *S*. *pombe* [57]. Studies in *S*. *cerevisiae* demonstrate that a γ-D68N mutation reduces GDP binding affinity [106]. In humans, an identical de novo *gtu1* mutation is associated with malformations of cortical development [88].

### Integrating information across databases

**Fig 3:**
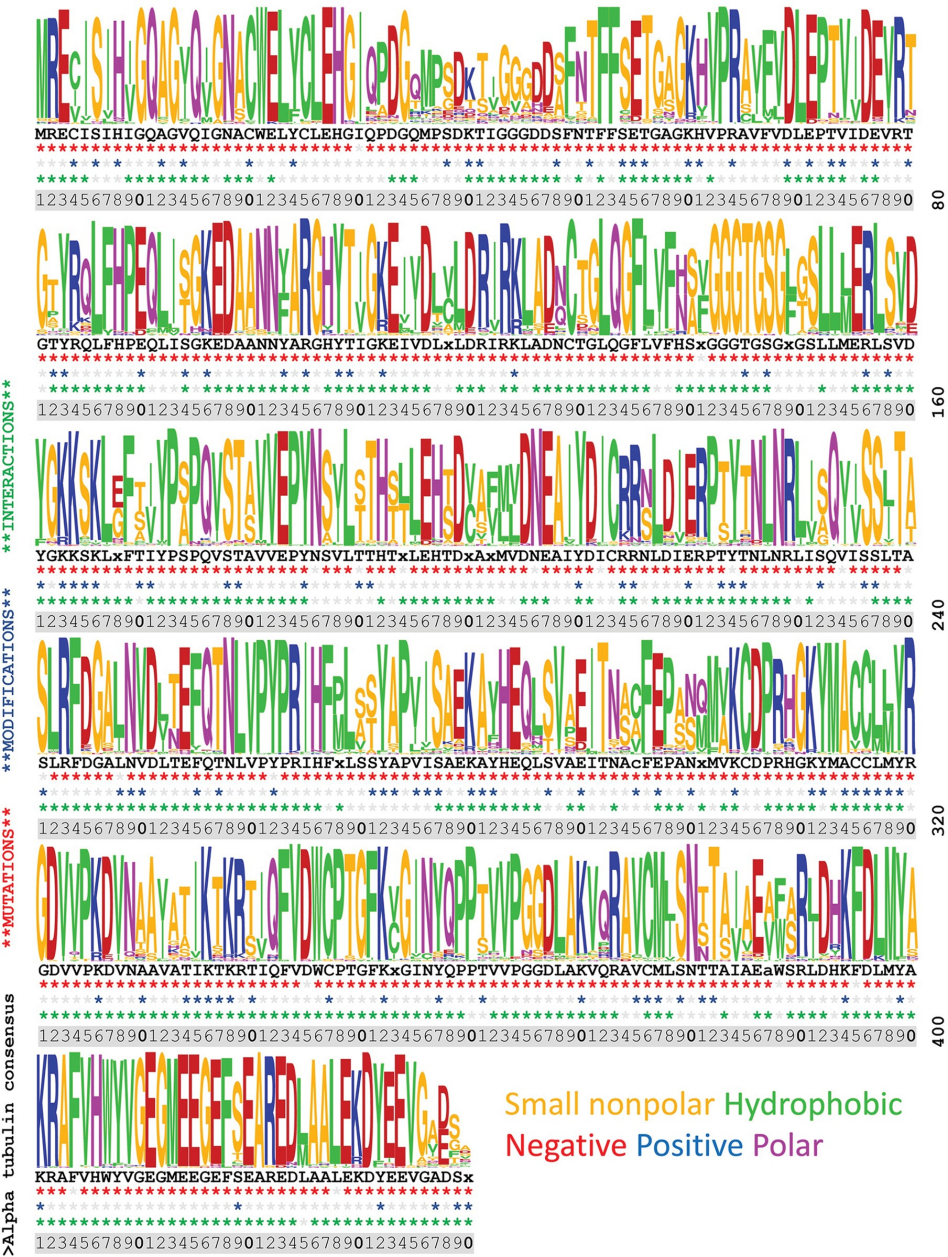
Sequence diversity, sites of mutations, modifications, and interactions in α-tubulin residues 1–440. Diversity is represented by a sequence logo graphical representation with the corresponding consensus sequence displayed below. An alignment of 90 sequences from organisms represented in the mutation database was used to create the image (see Methods for accession numbers). Amino acids that are represented by lowercase letters meet a lower statistical threshold as a consensus residue and “x” denotes positions that lack a clear consensus. For each position, three rows of asterisks are used to map intersections of indexed data: these indicate positions with documented mutations (red), sites of PTMs (blue); and sites of interaction (green).

**Fig 4:**
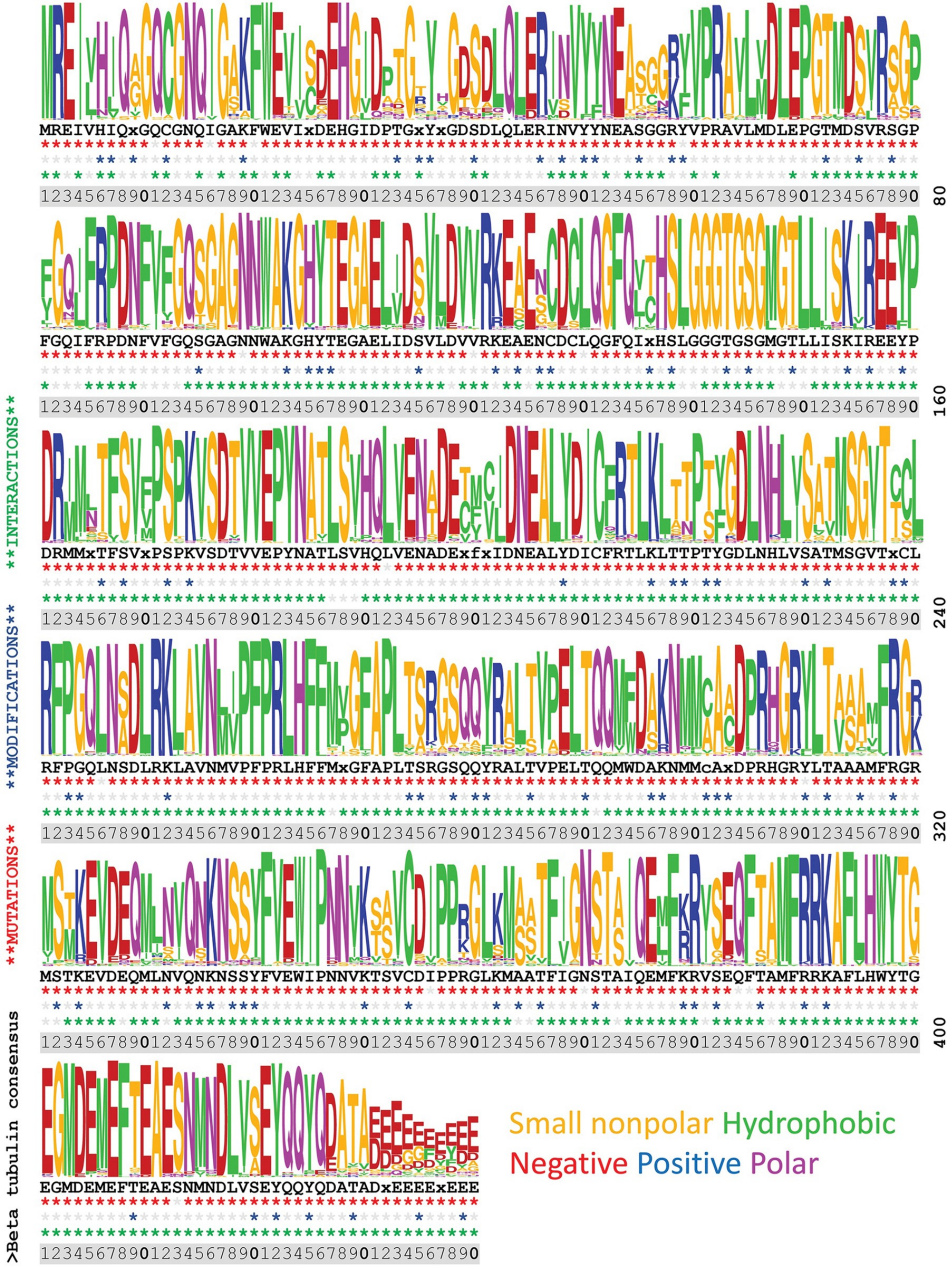
Sequence diversity, sites of mutations, modifications, and interactions in β-tubulin residues 1–440. An alignment of 90 representative β-tubulin sequences was used to create the image (see Methods for accession numbers). The details of this figure are identical to those for Fig 3.

**Fig 5:**
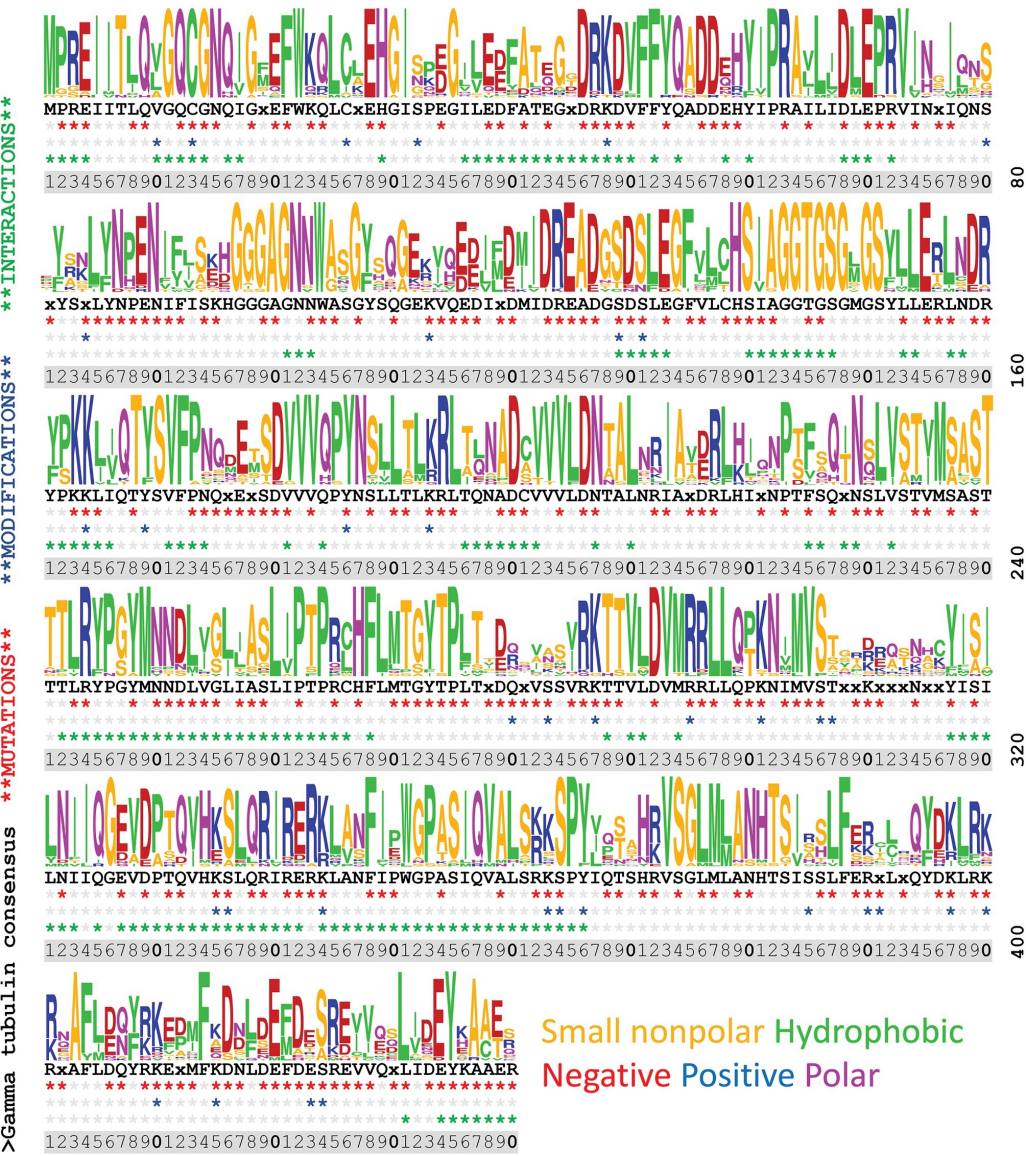
Sequence diversity, sites of mutations, modifications, and interactions in γ-tubulin residues 1–440. A Clustal Omega alignment of 25 γ-tubulin sequences from organisms represented in the mutation database was used to create the image (see Methods for accession numbers). The details of this figure are identical to those for Fig 3.

Sequence logos are typically used to graphically represent conservation within stretches of DNA or amino acid sequences [107]. Because tubulins have extraordinary homology, we used this analysis to illustrate sites of conservation and diversity along the full length of α-tubulin (**Fig 3**), β-tubulin (**Fig 4**), and γ-tubulin (**Fig 5**). These images were generated using the gap-deleted 90 amino acid Clustal alignments that were used to create the consensus sequences. The corresponding consensus sequence is displayed below the sequence logo and incidences of indexed mutations, PTMs, and interactions represented in the indexed entries to date are indicated for each position. The bulk of data defining PTMs represents information collected on yeast and mouse model organisms and from human cell cultures. The lack of an indexed modification in other species should not be taken as evidence that it does not occur in less well profiled eukaryotic tubulins. In the aggregate, there is nearly complete coverage of the tubulin sequence represented in the missense mutation databases, although not all positions are equally information dense. Importantly, data from systematic alanine scanning analysis and ectopic expression of heterologous proteins may not align with results from forward genetic screens and clinical datasets. Ultimately convergences represented in these datasets should be a starting point for mechanistic studies using genetic and biochemical methods, rather than an in-place, comprehensive explanation of function.

### Intersections of structural interfaces and missense mutations

Tubulinopathies are a collection of clinical conditions caused by de novo tubulin mutations that impair neuronal migration during development to cause severe brain malformations [103,108,109]. Expression of αR402, αG436 and αV409 missense mutations in budding yeast and mouse model organisms implicate impaired dynein or XMAP215 interactions as the defect underlying these tubulinopathies [110–112]. Although αR402 substituted tubulins assemble into microtubules, these do not support dynein motor activity and ectopic expression of R402C or R402H substituted α-tubulin disrupts cortical neuronal migration in the developing mouse brain [110]. In contrast, expression of G436R-substituted α-tubulin in budding yeast enhances dynein activity leading to spindle-positioning defects [112]. Lastly, αV409I and αV409A mutations reduce XMAP215/Stu2 affinity leading to increased microtubule dynamics [111]. In the aggregate, these genetic and biochemical studies indicate that changes to the microtubule surface alter its interactions with MAPs and motors.

Using a cryo-electron microscopy structure (3J1U) of the microtubule-binding domain of cytoplasmic dynein bound to microtubules [113], we identified tubulin residues within 6Å of dynein. This information was used to identify clinical mutations that alter human α- or β-tubulins at this interface (**Table 1**). In addition to well-recognized pathogenic substitutions at αR402, we identify 13 other sites in the dynein footprint that intersect with clinical conditions including lissencephaly, CFEOM, syndromic arthrogryposis multiplex congenita (AMC), hypomyelination with atrophy of the basal ganglia and cerebellum (H-ABC), and female infertility due to oocyte maturation defects. A variant implicated in risk of familial amyotrophic lateral sclerosis is also located within this interface. Additional tubulin clinical mutations are located at the Stu2 (XMAP215) protein interface, which partially intersects with the dynein footprint (not shown). Mutations of other loci, including the neuron-specific MAP doublecortin, also induce lissencephaly [114,115]. Doublecortin binds to a distinct site on microtubules from that occupied by dynein or kinesin, and we identify a second set of missense mutations and variants associated with neurological disorders at the tubulin-doublecortin interface (**Table 2**). This suggests impaired tubulin-doublecortin interaction may arise after changes to either protein to compromise neuron function. Importantly, the tubulin missense mutations may also impair interaction with other MAPs or motors as well to impair neuronal migration.

**Table 1:** Clinical variants at the dynein-tubulin interface.

Mutation	Phenotype	Reference
T110A	Tubulinopathies, tubulin α-1A	CV 625494
E113K	Tubulinopathies, tubulin α-1A	CV 625503
C402Y	Lissencephaly, Hirschsprung disease, tubulin α-1A	PM 23528852
R402C	Lissencephaly, tubulin α-1A	PM 29671837
R402C	Lissencephaly, tubulin α-1A	PM 18669490
R402C	Lissencephaly, tubulin α-1A	PM 25059107
R402C	Lissencephaly, tubulin α-1A	PM 20466733
R402C	Lissencephaly, tubulin α-1A	PM 18954413
R402C	Lissencephaly, tubulin α-1A	PM 17584854
R402H	Lissencephaly, tubulin α-1A	PM 29671837
R402H	Lissencephaly, tubulin α-1A	PM 25059107
R402H	Lissencephaly, tubulin α-1A	PM 17218254
R402H	Lissencephaly, tubulin α-1A	PM 20466733
R402H	Lissencephaly, tubulin α-1A	PM 17584854
R402H	Malformations of cortical development, tubulin α-1A	PM 35017693
R402L	Lissencephaly, tubulin α-1A	PM 18954413
R402L	Lissencephaly, tubulin α-1A	PM 22264709
R402S	Lissencephaly, tubulin α-1A	CV 160145
H406D	Congenital fibrosis of the extraocular muscles, tubulin α-1A	PM 33649541
H406Y	Fetal tubulinopathy, tubulin α-1A	PM 36403095
W407X	Familial amyotrophic lateral sclerosis association, tubulin α-4A	PM 25374358
V409A	Lissencephaly, tubulin α-1A	PM 25059107
V409I	Tubulinopathies, tubulin α-1A	CV 625504
E411G	Lissencephaly, tubulin α-1A	CV 620053
G412R	Syndromic arthrogryposis multiplex congenita, tubulin α-1A	PM 35686685
G416D	Lissencephaly, tubulin α-1A	CV 591001
G416S	Tubulinopathies, tubulin α-1A	CV 384538
N162K	Cortical dysplasia, tubulin β-2A	CV 127100
A163V	Cortical dysplasia, tubulin β-2A	CV 127101
G163R	Leukodystrophy, tubulin β-4A	CV 643798
T196A	Agenesis of the corpus callosum, tubulin β-3	PM 36403095
L253P	Cortical malformations, tubulin β-2B	PM 32570172
R262C	Extraocular congenital fibrosis syndrome, tubulin β-3	PM 20074521
R262H	Extraocular congenital fibrosis syndrome, tubulin β-3	PM 20074521
R262H	Extraocular congenital fibrosis syndrome, tubulin β-3	PM 24612975
R262H	Hypomyelination (H-ABC), tubulin β-4A	PM 24706558
R262H	Hypomyelinating leukoencephalopathy, tubulin β-4A	PM 24850488
R262H	Arthrogryposis multiplex congenita, tubulin β-2A	PM 28840640
R262H	Hypomyelination (H-ABC), tubulin β-4A	PM 24974158
R262Q	Defects in oocyte meiosis and female infertility, tubulin β-8	PM 26789871
R262W	Defects in oocyte meiosis and female infertility, tubulin β-8	PM 27273344
R262W	Defects in oocyte meiosis and female infertility, tubulin β-8	PM 36463079

Dynein motor footprint from PDB 3J1U: α-tubulin 104, 108–110, 112, 113, 402, 403, 405–417, 420 and β-tubulin 158, 162, 163, 196, 253, 261–264, 431, 434. **PM**: PubMed Identification Number; **CV**: ClinVar Number.

**Table 2:** Clinical variants at the doublecortin interface.

Mutation	Phenotype	Reference
E155D	Brain malformations, mirror movements, tubulin α-1A	PM 33413009
S158L	Fetal Tubulinopathy, tubulin α-1A	PM 36403095
S158L	Polymicrogyria like cortical dysplasia	PM 25059107
Y161D	Lissencephaly, pathogenic, tubulin α-1A	CV 160158
Y161H	Polymicrogyria, tubulin α-1A	PM 22948023
I196L	Tubulinopathies, tubulin α-1A	CV 625493
P263T	Lissencephaly, tubulin α-1A	PM 18669490
P263T	Cortical malformations, lissencephaly, tubulin α-1A	PM 17584854
R264C	Lissencephaly, tubulin α-1A	PM 29671837
R264C	Cerebellar dysplasia, tubulin α-1A	PM 28677066
R264C	Polymicrogyria, pachygyria, tubulin α-1A	PM 18728072
R264C	Lissencephaly, tubulin α-1A	PM 17218254
R264C	Cortical malformations, lissencephaly, tubulin α-1A	PM 17584854
R264G	Tubulinopathies, pathogenic, tubulin α-1A	CV 265283
R264H	Lissencephaly, tubulin α-1A	CV 287392
R264H	Microlissencephaly, tubulin α-1A	PM 25059107
R264P	Fetal Tubulinopathy, tubulin α-1A	PM 36403095
A333V	Polymicrogyria, tubulin α-1A	PM 23361065
K336R	Tubulinopathies, tubulin α-1A	CV 625496
V336E	Tubulinopathies, tubulin α-1A	CV 625514
M342V	Lissencephaly, tubulin α-1A	CV 160142
Q342P	Tubulinopathies, tubulin α-1A	CV 449112
A348D	Tubulinopathies, tubulin α-1A	CV 427202
A348V	Lissencephaly, tubulin α-1A	CV 160143
K430N	Familial ALS association, tubulin α-4A	PM 25374358
D431G	Lissencephaly, tubulin α-1A	PQ 2552055004
V435A	Lissencephaly, tubulin α-1A	CV 437120
D438N	Sporadic ALS risk, tubulin α-4AA	PM 25893256
L106F	Cortical dysplasia, other brain malformations, tubulin β	CV 807517
T106M	Cortical dysplasia, tubulin β-3	CV 265335
T106M	Leukodystrophy, tubulin β-4A	CV 267774
T106R	Leukodystrophy, tubulin β-4A	CV 139453
P110T	Leukodystrophy, hypomyelinating, tubulin β-4A	CV 267775
P173L	Malformation of cortical development, tubulin β-2B	PM 25059107
N175S	Cortical dysplasia, brain malformations, tubulin β-3	CV 160196
V175L	Hydrocephalus, Walker–Warburg Syndrome, tubulin β-3	PM 29187032
E205K	Malformation of cortical development, tubulin β-3	PM 20829227
Q206P	Cortical dysplasia, brain malformations, tubulin β-2A	CV 217024
A302T	Extraocular congenital fibrosis syndrome, tubulin β-3	PM 20074521
A302T	Infantile esotropia, tubulin β-3	PM 35765833
A302T	Fibrosis of extraocular muscles, tubulin β-3	CV 6964
A302V	Malformation of cortical development, tubulin β-3	PM 20829227
A302V	Cortical dysplasia, tubulin β-3	CV 30275
M302L	Leukodystrophy, tubulin β-4A	CV 537237
R306C	Cortical dysplasia, tubulin β-2A	CV 521900
V306I	Leukodystrophy, tubulin β-4A	CV 217025
P307L	Cortical dysplasia, tubulin β	CV 438586
R307H	Leukodystrophy, tubulin β-4A	CV 265314
A308V	Cortical dysplasia, tubulin β-2A	CV 800924
M308L	Leukodystrophy, β-4A	CV 537237
R380C	Congenital fibrosis of the extraocular muscles, tubulin β-3	PM 20074521
R380C	Cortical malformations, tubulin β-2B	PM 23361065
R380C	Cerebellar dysplasia, tubulin β-3	PM 28677066
R380C	Cerebellar dysplasia, tubulin β-2B	PM 28677066
R380S	Cortical malformations, tubulin β-2B	PM 23361065
R390L	Bilateral ventriculomegaly, tubulin β-2B	PM 36403095
R390Q	Uner Tan syndrome, tubulin β-2B	PM 28013290
R391C	Leber congenital amaurosis, tubulin β-4B	PM 29198720
R391C	Ophthalmologic and hearing impairments, tubulin β-4B	PM 35240325
R391C	Cortical dysplasia, tubulin β-2A	CV 521900
R391H	Hypomyelination, tubulin β-4B	PM 25085639
R391H	Leber congenital amaurosis, tubulin β-4B	PM 29198720
R391L	Leukodystrophy, tubulin β-4A	CV 419697
H396Y	Tubulinopathy, tubulin β-2A	PM 32571897
A397S	Leukodystrophy, tubulin β-4A	CV 807519
A397T	Leukodystrophy, tubulin β-4A	CV 267785
C399W	Leukodystrophy, tubulin β-4A	CV 453295
C399Y	Leukodystrophy, tubulin β-4A	CV 267786
D400E	Leukodystrophy, tubulin β-4A	CV 807518
E401K	Microcephaly, tubulin β	PM 23246003
E401K	Microcephaly, tubulin β	PM 30738969
E401K	Cortical dysplasia, tubulin β	CV 127191
A403S	Leukodystrophy, tubulin β-4A	CV 807519
A403T	Leukodystrophy, tubulin β-4A	CV 267785

Doublecortin footprint from PDB 6RF2 and 6RF8: α-tubulin 155, 158–163, 166, 195–197, 262–264, 333, 335–343, 345, 348, 430, 431, 434, 435, 438–441 and β-tubulin 94, 95, 103, 106–108, 110, 111, 114, 173–175, 205, 206, 209, 213, 301–304, 306–308, 376, 380, 383, 384, 390–392, 395–397, 399–405, 426. **CV**: ClinVar; **PM**: PubMed; **PQ**: Proquest.

### Intersections of missense mutations and modification sites

Organizing the PTM dataset revealed that many modification sites intersect with missense mutations in the genetic database: loss of key amino acids (serine, threonine, tyrosine, arginine, cysteine, and lysine) prevents dynamic regulation by reversible modification. These convergences raise the possibility that the inability of variants to be actively regulated during microtubule-dependent processes is a significant contributing factor to the observed defects. A recent study used *Drosophila* as a model organism to investigate the role of acetylation of α-tubulin K394, a PTM observed across many species in many α-tubulin isotypes [116]. Introduction of a PTM-blocking K394R mutation in the primary *Drosophila* α-tubulin gene (84B) caused decreased microtubule stability and impaired neuron morphogenesis. We realized that the NIH ClinVar database describes a case of lissencephaly (864867) associated with a K394N substitution to TUBA1A and MS data verifies that K394 is acetylated in human TUBA1A. Although the ClinVar entry is annotated as of “uncertain significance” this substitution will ablate K394 acetylation, which may lead to parallel developmental defects to those described in the *Drosophila* study. Additional missense mutations eliminate sites of PTMs in α- and β-tubulins in tubulinopathies and other clinical conditions. **Table 3** lists examples of mutations that ablate sites of PTMs in α-based tubulinopathies. Among these, αT56 mutations eliminate phosphorylation at this site [97]. Notably, although loss of αT56 also leads to developmental defects in *Arabidopsis* and *Oryza* [80,117], information on tubulin PTMs in land plants is much more limited or altogether absent.

**Table 3:** α-tubulin 1A mutations ablate ptm sites in tubulinopathies.

Mutation	PTM	Phenotype	Reference
S54N	PHO	Cerebellar dysplasia	PM 28677066
S54N	PHO	Tubulinopathies	CV 625506
S54R	PHO	Lissencephaly	CV 160150
T56M	PHO	Fetal tubulinopathy	PM 36403095
T56M	PHO	Lissencephaly	CV 625513
T56M	PHO	Microlissencephaly	PM 25059107
T56R	PHO	Cerebellar dysplasia	PM 33165829
K60N	ACE, UBI, SMO	Lissencephaly	CV 632599
T73A	PHO	Fetal tubulinopathy	PM 36403095
S158L	PHO	Fetal tubulinopathy	PM 36403095
S158L	PHO	Tubulinopathies,	CV 625517
S158L	PHO	Polymicrogyria	PM 25059107
Y161D	PHO	Lissencephaly	CV 160158
Y161H	PHO	Tubulinopathies	CV 625477
Y161H	PHO	Polymicrogyria	PM 22948023
Y210C	PHO	Epileptic encephalopathy	PM 35892608
Y210C	PHO	Tubulinopathies	CV 625474
Y210C	PHO	Lissencephaly	PM 21403111
K326N	ACE, UBI, SMO	Tubulinopathies	CV 625487
K326N	ACE, UBI, SMO	Microlissencephaly	PM 25059107
K336R	ACE, UBI, SMO	Tubulinopathies	CV 625496
K352M	ACE, UBI, SMO	Tubulinopathies	CV 521592
K394N	ACE, UBI, SMO	Lissencephaly	CV 864867
S419L	PHO	Lissencephaly	CV 7074
S419L	PHO	Lissencephaly	PM 17584854

**PHO**: Phosphorylation; **ACE**: Acetylation; **UBI**: Ubiquitination; **SMO**: SUMOylation.

**PM**: PMID number; **CV**: ClinVar database number.

By cross-correlating missense data and sites of PTMs, we realized that there are significant losses of modifiable sites in a collection of mutant β-tubulin genes isolated from breast cancer tumor samples. The original report describes mutations in βI-, βIIA-, or βIVB-tubulin genes that make these tubulins more like the βIII-tubulin isotype which is overexpressed in aggressive and metastatic cancers [118]. In most cases, there are multiple changes that convert between 2 and 9 amino acids in βI-, βIIA-, or βIVB-tubulin isotypes to residues found in βIII-tubulin. The paper also describes βIII-tubulin mutations that convert conserved residues to amino acids characteristic of βI-, βIIA-, or βIVB-tubulin isotypes. Of the 21 mutant isotypes documented in the report, 17 harbor changes that eliminate at least one PTM site (**Table 4**). Notably, many samples harbor changes that eliminate β-tubulin C239, which removes the ability of this position to be palmitoylated and nitrosylated. Palmitoylation (addition of a 16-carbon fatty acid) modulates membrane association, subcellular localization, and stability [119] whereas nitrosylation influences the capacity of tubulin to polymerize [120,121].

**Table 4:** Diverse tubulin mutations in breast cancer isolates eliminate sites of PTMs.

Type	Substitution(s)	Lost Modification
βI	**C239L**, I152T, L240P, R241P, L273P	Nitrosylation/palmitoylation
βI	**C239L**, **T237H**, **T238H**, L240P, R241P	Nitrosylation/palmitoylation, phosphorylation
βI	**C239Y**	Nitrosylation/palmitoylation
βI	**C239P**, L240P and R241P	Nitrosylation/palmitoylation
βI	**T238H**, **C239L**, L240P, and R241P	Nitrosylation/palmitoylation, phosphorylation
βI	**C239L**, L240P and R241P	Nitrosylation/palmitoylation
βI	D177G, **C239R**	Nitrosylation/palmitoylation
βI	I155V, **K324R**, L331H	Acetylation/Ubiquitination
βI	L112P, **T351V**, A364S, V365	Phosphorylation
βI	**T33S**, **T35N**	Phosphorylation
βIIA	E45D, N48S, V64I, V170M, C201S, A296S, V316I	None
βIII	A275T	None
βIII	V180A, **T315A**, A332N, I333V, **S335N**, V351T, **S365A**, I374T	Phosphorylation
βIII	I189V, A218T, A275S, Q291R, **K297R**, R320P, **S364A**, **T386S**	Acetylation/ubiquitination, phosphorylation
βIII	V170M	None
βIII	D249E, M293V, **T315A**, A332N, I333V, **S335N**, V351T, A352V, **S364A**, **S365V**	Phosphorylation
βIII	**T166A**, I189V, **S239C**, **T315A**, A332N, I333V, **S335N**, V351T	Phosphorylation
βIVB	P171L, **C239R**	Palmitoylation
βIVB	V333I, N332A, N335S, **T351V**, A365S	Phosphorylation
βIVB	L44P, **S275A**, Q292R, A315T, V333I, N332A, N335S, **T351V**, A365S	Phosphorylation
βIVB	E45D, N48S, V64I	None

All data is from the study by Wang et al. (2017) [118] Protoplasma Bolded missense mutations eliminate PTMs in isotype genes of specific breast cancer patients.

### Other applications of this granular data

**Fig 6:**
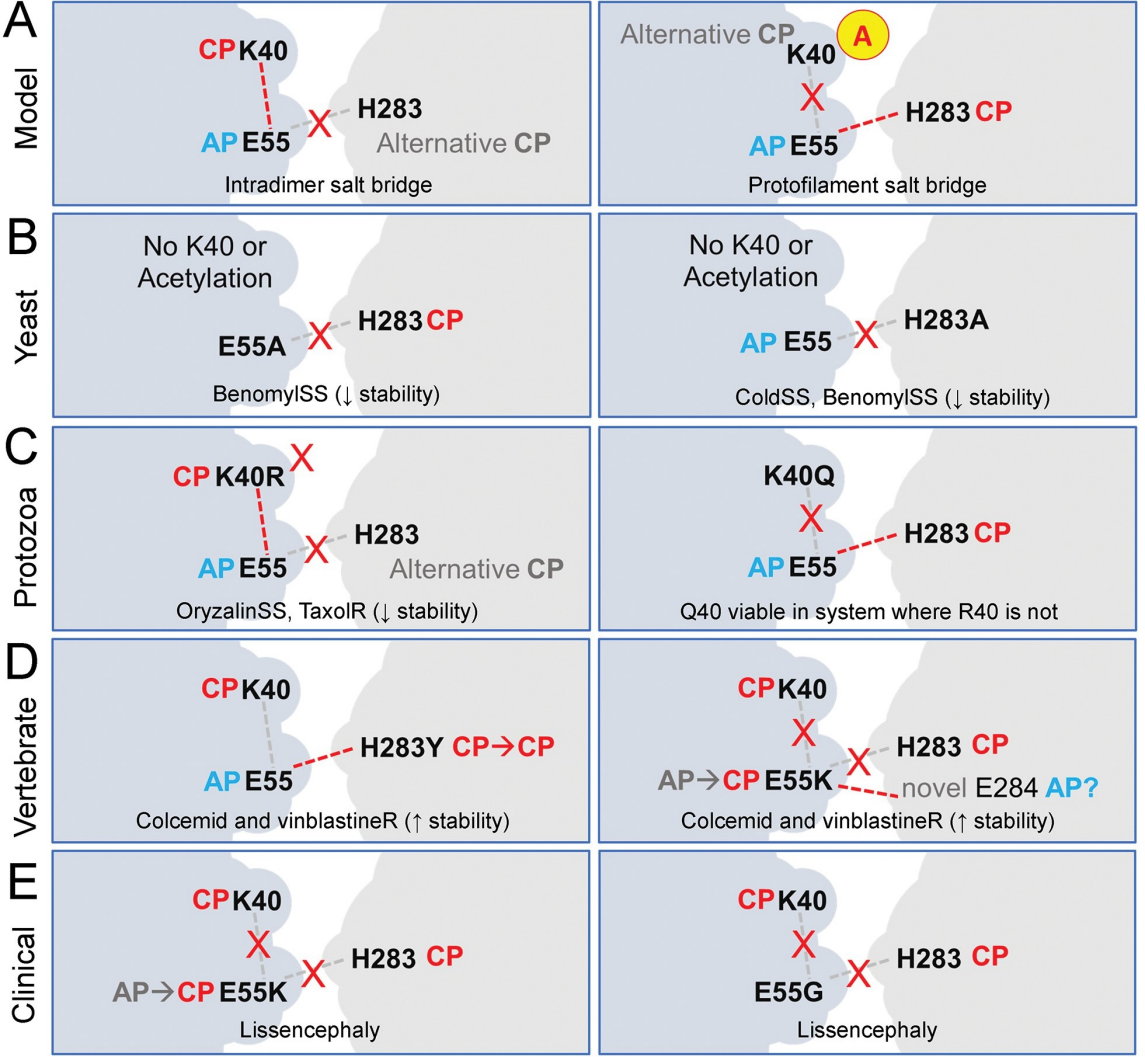
Genetic evidence for a proposed model of K40 acetylation and microtubule stability. (A) Molecular dynamics simulations by the Nogales group suggest that αK40 acetylation increases the likelihood that αE55 (anionic partner, AP) forms a salt bridge with αH283 (cationic partner, CP) in the adjacent dimer to increase microtubule stability. In this model, when αK40 is unacetylated, it forms an internal salt bridge with αE55 sequestering it from interaction with αH283. (B) In budding yeast, alanine substitutions at αE55 and αH283 cause supersensitivity (ss) to the microtubule-disrupting drug benomyl, in-line with loss of the protofilament-spanning salt bridge causing reduced microtubule stability. (C) Replacement of lysine with arginine, an amino acid that can form salt bridges as a cationic partner but cannot be acetylated, makes *Tetrahymena* hypersensitive to microtubule disruption and less sensitive to Taxol, a stabilizing drug. This phenotype is consistent with reduced stability microtubules when αE55 is sequestered. Substitution with glutamine (an acetylation mimetic) but not arginine is recovered in the single conventional α-tubulin gene in *Toxoplasma*, illustrating that the cross-filament salt bridge is essential for microtubule stability. (D) CHO cell mutations H283Y and E55K confer resistance to vinblastine and colcemid. These substitutions disrupt the salt bridge between αE55 and αH283 in adjacent protofilaments but would likely act to improve protofilament affinity. The Y283 substitution may still form salt bridges or may participate in novel polar interactions across protofilaments. If correctly positioned, K55 could serve as a cationic partner to form a novel salt bridge with E284. (E) E55K and E55G substitutions documented in cases of lissencephaly would disrupt both proposed salt bridges.

An increasing number of papers use computational modeling to explore tubulin function. Ideally, their conclusions should be tested by subsequent biochemical studies. An additional approach would be to assess whether in silico findings are consistent with available genetic (phenotypic) data. We illustrate this by using indexed missense mutations to explore the consequence of substitutions to residues that have been implicated in microtubule-stabilizing interactions. Acetylated αK40 microtubules are resilient to mechanical stresses and drug treatments that cause non-acetylated microtubules to break or disassemble. K40 is located within the α-tubulin H1’-S2 loop (also known as the N, P37 to D47, or K40 loop) that is required for lateral contacts between protofilaments [36,122]. A proposed mechanism for α-tubulin K40 acetylation to increase microtubule stability invokes alternative formation of exclusive salt bridge pairs [36]. Salt bridges are formed by the interaction of anionic residues (glutamic acid or aspartic acid) with cationic partners (lysine, arginine, histidine, or sometimes serine or tyrosine). Bridges arise when partners are sufficiently close to each other to facilitate electrostatic attraction [123]. Computational modeling has suggested that unacetylated αK40 forms a salt bridge with αE55 within the α-tubulin monomer (**Fig 6**). Acetylation reduces the likelihood that αK40 sequesters αE55, leaving αE55 available to form a salt bridge with αH283 in the M loop of the adjacent protofilament to increase microtubule stability [122]. Existing substitutions to αK40, αE55 and αH283 expose biochemical properties of these residues in microtubule stability. Although budding yeast does not acetylate H1’-S2 loop lysines, E55 or H283 substitutions cause increased sensitivity to the cold and/or benomyl-induced microtubule-disruption [59], consistent with reduced microtubule stability. In the protozoan ciliate *Tetrahymena*, an engineered K40R substitution replaces lysine with an amino acid that preserves charge and salt bridge forming capacity but cannot be acetylated. αK40R *Tetrahymena* have increased susceptibility to microtubule disruption with dinitroanilines and increased resistance to the microtubule stabilizing drug paclitaxel [124]. This is consistent with the model that E55 sequestration inhibits it from forming microtubule-stabilizing interactions with H283. Although researchers were unable to recover a K40R substitution in α-tubulin in the protozoan parasite *Toxoplasma gondii*, they were able to introduce the K40Q acetylation mimetic which likely promotes microtubule stability [125]. Studies in CHO cells identify H283Y and E55K mutations as conferring resistance to both vinblastine and colcemid [126]. Since these drugs bind to distinct sites, resistance likely increases microtubule stability. The αY283 substitution may also form a salt bridge with αE55 or may participate in a novel polar interaction with the adjacent protofilament. The E55K substitution replaces an anionic partner with a cationic one to increase stability. One possible explanation is that a novel salt bridge forms between αK55 and αE284 in the adjacent protofilament. E55K [81] and E55G (ClinVar 212491) substitutions to α-tubulin 1A have been documented in cases of lissencephaly, highlighting a critical role for E55 in microtubule function. Importantly, expanded biochemical and computational studies informed by this collection of mutant phenotypes are useful to refine the model as well as to augment other computational studies.

## Discussion

Tubulins are an ancient, highly conserved family of universal and essential proteins in eukaryotes [127]. Tubulin conservation is dictated by numerous functional constraints including essential GTP binding and assembly interfaces. In addition, both luminal and outer microtubule surfaces interact with essential proteins including MAPs, microtubule inner proteins (MIPs) and microtubule motors [128]. The greatest sequence diversity is observed among members of the δ-, ε- and ζ-tubulin families which have been lost from some eukaryotes and may therefore be less functionally constrained than the universal α-, β- and γ-tubulins.

Given the extraordinarily large volume of available information describing tubulins in diverse eukaryotes, extensive indexing of granular datasets reveals previously unappreciated convergent and divergent traits. Tubulin is uniquely suited for this analysis because it is a universal and essential protein in eukaryotes and is subject to strong sequence constraints. The expanded tubulin database described here collects and indexes detailed information that describes variants and missense mutations, PTMs, and amino acid interactions. We developed a universal numbering system to identify correspondences between equivalent positions. This indexing has been used to correlate information across species and tubulin isotypes for the α-, β- and γ-tubulin families. Although not all apparent correlations will be significant, mapping convergent positions highlights areas for future investigation that will help us better understand tubulin function.

Missense mutations or variants may alter local amino acid interactions to influence tubulin function. Changes to the proximal environment influence properties of the tubulin subunit, and protein-protein interactions between subunits and with associated proteins. In this paper as well as our previous study [79] we identify noteworthy examples of parallel mutations that arise in different isotypes, species, or contexts that may reflect the same or similar underlying biochemical changes to tubulin. While it is interesting to detect convergent missense mutations, it is critical for biochemists to explore the effects of substitutions on tubulin behavior. Not all parallel mutations function identically: although the β-tubulin missense mutation T238A slows microtubule shrinking in both *S*. *cerevisiae* and *H*. *sapiens* microtubules, the mechanism underlying this effect differs between species. *S*. *cerevisiae* microtubules containing T238A β-tubulin have reduced disassembly due to suppression of a conformational change initiated by polymerization-dependent GTP hydrolysis [129]. In the case of isotype-specific recombinant human tubulin, βT238A microtubules polymerize more rapidly than wild-type microtubules and the substitution does not appear to influence the conformational change induced by GTP hydrolysis [130]. Since tubulins from different species are distinguished by some number of amino acid differences (budding yeast and human β-tubulins have ~25% distinct amino acids), the effect of a substitution may be modulated by other amino acid differences.

With large datasets, readers may question whether the intersections we note are cherry-picked examples that are consistent with a “just so” story we can craft. No correlations should be taken as an explanation of mechanism without further investigation. It is critical that researchers use the Tubulin Database as a resource to identify apparent correspondences or divergences to further explore underlying tubulin biochemistry. We were delighted by the response to our original tubulin mutation database [79] and appreciate the encouragement, feedback, and corrections communicated to us by colleagues. The database expansion described here substantially enlarges this resource. In addition to future updates and corrections to the tubulin database, we can imagine further expansion of additional linked resources. We welcome feedback from and collaborations with researchers with expertise in interacting proteins, such as microtubule motors, who would like to construct complementary resources for these proteins.

## Materials and methods

### 1. Creating a universal tubulin numbering system

In order to assemble a diverse set of tubulin amino acid sequences, we used the UniProt (www.uniprot.org) and NCBI (www.ncbi.nlm.nih.gov/protein) databases to collect available tubulin isotype sequences for organisms such as represented in our genetic database [131,132]. We collected isotype sequences from land plant, nematode, fungus, protozoan and vertebrate organisms. Full-length α-, β-, or γ-tubulins were aligned in Clustal Omega (www.clustal.org/omega) and gaps created by uncommon insertions were removed from the alignment [133–135]. For this analysis, we used 90 unique α- or β-tubulins, and 21 γ-tubulins as follows. α-tubulin sequences: A0A5B9TE06, A0A5B9T7A6, A0A5B9T794, A0A5B9T5X2, A0A5B9T627, A0A5B9TEC1, Q65C79, Q65C78, A0A4U6STT5, A0A4U6W1W4, A0A4U6T6L3, D5MRD7, D5MRD8, D5MRD9, D5MRE0, Q8WRT6, P41351, I7LY16, Q23WP5, Q22CD2, P06603, P06604, P06605, P06606, Q9VED6, O22347, O22348, O22349, O18688, P34690, P91910, G5EDD4, Q19490, P91873, O18154, P52274, Q20221, Q71U36, P68363, Q9BQE3, P0DPH7, P0DPH8, Q6PEY2, P68366, Q9NY65, A6NHL2, O22661, O22660, A0A059T4F2, A0A059T4U6, A0A059T4Y0, A0A059T4F4, A0A059T464, P11139, B9DGT7, Q56WH1, Q0WV25, B9DHQ0, P29511, P28752, Q53M52, P68362, P68361, P68365, P09204, P09205, Q9DFR8, Q9DD79, Q9DFR7, Q9DFR9, Q78C10, Q9YHW2, Q9YHW1, Q9DFT3, Q7XYS1, Q84KQ3, A0A125YFV3, P04688, P04689, P09733, P09734, P68369, P05213, P68373, P05214, P68368, Q9JJZ2, A0A125YQG0, A0A125YTP7, and A0A125YQ52. β-tubulin sequences: A0A385HDR4, P12411, Q56YW9, Q9ASR0, P24636, P29513, P29514, P29515, P29516, P29517, O17921, P12456, P52275, P41937, G5EF01, Q18817, Q9DD57, P04690, P69893, A0A125YTB7, Q84KQ2, Q7XYS0, Q24560, P61857, P08841, Q9VAX7, Q9VRX3, Q9ZPP0, Q9ZPN9, Q9ZPN8, Q9ZPN7, Q9N2N6, C0L7F0, C0L7F2, C0L7F1, P07437, Q9H4B7, Q13885, Q9BVA1, Q13509, P04350, P68371, Q9BUF5, Q3ZCM7, A6NNZ2, A0A5B9T5X9, A0A5B9T5T1, A2AQ07, Q7TMM9, Q9CWF2, Q9ERD7, Q9D6F9, P68372, P99024, Q922F4, P36221, Q78C12, Q9DFT5, Q9DFT6, Q43594, Q8H7U1, Q40665, P45960, P46265, Q76FS3, P37832, Q76FS2, P02557, P05219, Q65C77, Q65C76, A0A4U6T2L9, A0A4V6D9T7, A0A4U6UB67, A0A4U6SU51, A0A4U6V2J9, A0A4U6WIH1, P41352, Q24D63, Q24D62, I6U4Q6, Q22ED2, A0A125YYZ6, A0A125YJU4, A0A125YWG5, P10653, P10874, A0A226BMV8, A0A226BIW8, and E2RFJ7. γ-tubulin sequences: P38557, P38558, P34475, Q39582, G3HLY0, P23257, P42271, A3F2R1, P23258, Q9NRH3, P83887, Q8VCK3, O49068, P53378, P25295, A0A4U6, O00849, S8F111, P18695, O96769, and Q9TYH0.

Edited alignments were submitted to Emboss (www.ebi.ac.uk/Tools/msa/emboss_cons/) to generate consensus sequences and to determine pairwise conservation between tubulins [136]. Individual tubulins were each aligned to the consensus sequence to adjust the numbering to correspond to the consensus (**S1 Table**). Due to heterogeneity in the CTTs of tubulins, all consensus sequences are truncated at 440 amino acids. WebLogo tools (https://weblogo.berkeley.edu/logo.cgi) were used to graphically represent identity and diversity at individual positions in the edited alignments [137]. Amino acids were colored according to Lesk [138]: orange = small nonpolar (G, A, S, T); green = hydrophobic (C, V, I, L, P, F, Y, M, W); magenta = polar (N, Q, H); red = negatively charged (D, E); and blue = positively charged (K, R). This coloring is carried over to the mutation web data tables. We did not create a universal consensus for δ-, ε- and ζ- tubulin families because alignments of these proteins reveal significantly larger insertions that vary in location and sequence.

### 2. Indexing post-translational modification data

We collected tubulin PTM information using predictive and mass spectrometry (MS) databases to achieve granular detail specifying species, isotype, amino acid and modification type. Information describing tubulin PTMs was collected from the GlyGen (glygen.org); iPTMnet (research.bioinformatics.udel.edu/iptmnet); PhosphoSitePlus (phosphosite.org); SwissPalm (swisspalm.org) and YAAM (http://yaam.ifc.unam.mx) databases [139–145]. The tubulin PTM data is indexed by universal amino acid position for individual tubulin families and entries include links to the original data resources.

### 3. Collecting information from tubulin structures

We reviewed over 300 tubulin-containing structures from the RCSB Protein Databank (www.rcsb.org/) [146]. After sorting these into categories such as small molecule-bound tubulins or microtubules with associated proteins, we chose representative structures to capture tubulin residues that contribute to protein or ligand interactions. PyMol (https://pymol.org) was used to select amino acids that are within 6 Å of key structures (e.g., bound GTP, MTAs, associated proteins) [147]. The extraction tool was used with an Excel plugin to copy information. Final entries include hyperlinks to the structure and reference.

### 4. Updating and expanding the tubulin mutation database

We continue to use terms such as “tubulin mutation” and “mutant tubulin” in Google and PubMed searches to identify new papers that describe tubulin point mutations to index. Importantly, we also query “tubulin variant” to find relevant articles that concern clinical conditions. In addition to integrating data described in publications, we collected information from the NCBI ClinVar (www.ncbi.nlm.nih.gov/clinvar/) [148,149] and BioMuta (https://hive.biochemistry.gwu.edu/biomuta) [150] databases to incorporate human single nucleotide polymorphism (SNP) data. We strove to standardize nomenclature and symbols based on guidelines described at http://varnomen.hgvs.org/bg-material/basics/, particularly with respect to human findings. Specifically, human mutations are somatic changes to tubulin genes that cause substitutions in some tissues (cancer cells, tubulinopathies). Tubulin genes that specify proteins that differ from reference sequences and may be associated with increased risk of disease are termed variants. Although there is some redundancy between entries collected from ClinVar or BioMuta and data gathered from publications, to prevent misinterpretation or clerical errors, we did not compress information from different sources into single entries.

### 5. Creating and Integrating tubulin datasets

Using publicly available data we assembled tables that document: (1) point mutations to tubulin; (2) PTMs to tubulins; (3) local ligand interactions; and (4) local interactions that contribute to protein subunit structure and protein-protein interactions. All datasets incorporate hyperlinks to relevant publications and database information and were transformed into web-based resources using https://datatables.net/ tools with JavaScript, HTML and CSS. DataTables were edited using Notepad++. Intersecting data is cross-referenced in synthesis tables. Phylogenetic analysis locates additional unusual protozoan tubulins as deep branching members of the α-tubulin (κ-), β-tubulin (θ, ι) or ζ-tubulin (η) families [4]. This information was used to place relevant information for these proteins within appropriate tubulin family tables.

## Supporting information

S1 Table Consensus amino acid sequences for α-, β-, and γ-tubulins used to establish the universal tubulin numbering system. (DOCX)
