# Supplementary material for: The tubulin database: Linking mutations, modifications, ligands and local interactions
Source: PLoS One. 2023 Dec 8;18(12):e0295279. doi: 10.1371/journal.pone.0295279 (PMC10707541; doi:10.1371/journal.pone.0295279)
Supplement: S1 Table — (DOCX) [file pone.0295279.s001.docx]

| **Table S1: Consensus sequences for universal tubulin numbering** | |
| --- | --- |
| Alpha tubulin consensus | MRECISIHIGQAGVQIGNACWELYCLEHGIQPDGQMPSDKTIGGGDDSFNTFFSETGAGKHVPRAVFVDLEPTVIDEVRTGTYRQLFHPEQLISGKEDAANNYARGHYTIGKEIVDLxLDRIRKLADNCTGLQGFLVFHSxGGGTGSGxGSLLMERLSVDYGKKSKLxFTIYPSPQVSTAVVEPYNSVLTTHTxLEHTDxAxMVDNEAIYDICRRNLDIERPTYTNLNRLISQVISSLTASLRFDGALNVDLTEFQTNLVPYPRIHFxLSSYAPVISAEKAYHEQLSVAEITNAcFEPANxMVKCDPRHGKYMACCLMYRGDVVPKDVNAAVATIKTKRTIQFVDWCPTGFKxGINYQPPTVVPGGDLAKVQRAVCMLSNTTAIAEaWSRLDHKFDLMYAKRAFVHWYVGEGMEEGEFSEAREDLAALEKDYEEVGADSx |
| Beta tubulin consensus | MREIVHIQxGQCGNQIGAKFWEVIxDEHGIDPTGxYxGDSDLQLERINVYYNEASGGRYVPRAVLMDLEPGTMDSVRSGPFGQIFRPDNFVFGQSGAGNNWAKGHYTEGAELIDSVLDVVRKEAENCDCLQGFQIxHSLGGGTGSGMGTLLISKIREEYPDRMMxTFSVxPSPKVSDTVVEPYNATLSVHQLVENADExfxIDNEALYDICFRTLKLTTPTYGDLNHLVSATMSGVTxCLRFPGQLNSDLRKLAVNMVPFPRLHFFMxGFAPLTSRGSQQYRALTVPELTQQMWDAKNMMcAxDPRHGRYLTAAAMFRGRMSTKEVDEQMLNVQNKNSSYFVEWIPNNVKTSVCDIPPRGLKMAATFIGNSTAIQEMFKRVSEQFTAMFRRKAFLHWYTGEGMDEMEFTEAESNMNDLVSEYQQYQDATADxEEEExEEE |
| Gamma tubulin consensus | MPREIITLQVGQCGNQIGxEFWKQLCxEHGISPEGILEDFATEGxDRKDVFFYQADDEHYIPRAILIDLEPRVINxIQNSxYSxLYNPENIFISKHGGGAGNNWASGYSQGEKVQEDIxDMIDREADGSDSLEGFVLCHSIAGGTGSGMGSYLLERLNDRYPKKLIQTYSVFPNQxExSDVVVQPYNSLLTLKRLTQNADCVVVLDNTALNRIAxDRLHIxNPTFSQxNSLVSTVMSASTTTLRYPGYMNNDLVGLIASLIPTPRCHFLMTGYTPLTxDQxVSSVRKTTVLDVMRRLLQPKNIMVSTxxKxxxNxxYISILNIIQGEVDPTQVHKSLQRIRERKLANFIPWGPASIQVALSRKSPYIQTSHRVSGLMLANHTSISSLFERxLxQYDKLRKRxAFLDQYRKExMFKDNLDEFDESREVVQxLIDEYKAAER |
| Amino acids that are represented by lowercase letters meet a lower threshold as a consensus residue and x is used to denote positions where there is no consensus at that location.  Due to the heterogeneity in the carboxy-terminal tails of tubulins, all consensus sequences are truncated at 400 amino acids.  The much greater heterogeneity in sequences and indels for δ-, ε- and ζ- tubulins precludes developing universal sequence numbering for these families. | |
